# Supplementary material for: Spatial variation of parrotfish assemblages at oceanic islands in the western Caribbean: evidence of indirect effects of fishing?
Source: PeerJ. 2022 Nov 28;10:e14178. doi: 10.7717/peerj.14178 (PMC9744149; doi:10.7717/peerj.14178)
Supplement: Supplemental Information 1 — Year, location and depht (m) range per station. Stations were located in coral reefs bottoms and some nearby seagrass medows (*). [file peerj-10-14178-s001.pdf]

Table S1. Video-stations surveyed. Year, location and depht (m) range per station. Stations were located in coral reefs bottoms and some nearby seagrass medows (*).

Year	Locality	Station	Latitude	Longitude	Depth (m)
2018	Bolivar (BOL)	1	12.39201	-81.46647	8-15
		2	12.39619	-81.47939	15-30
		3	12.41922	-81.47945	8-15
		4	12.4348	-81.49897	15-30
		5	12.41774	-81.47878	3-8
		6	12.39331	-81.45147	8-15
		7	12.41336	-81.47147	8-15
		8	12.44653	-81.49506	15-30
		9	12.38563	-81.45908	15-30
		10	12.38937	-81.45805	3-8
		11	12.39575	-81.46768	3-8
		12	12.41169	-81.48862	15-30
		13	12.39856	-81.47815	3-8
		14	12.40249	-81.48354	8-15
		15	12.41905	-81.49595	15-30
		16	12.45195	-81.4883	3-8
	San Andrés (SA)	1	12.595958	-81.718196	15-30
		2	12.594199	-81.690987	3-8
		3	12.49833564	-81.71440905	15-30
		4	12.54725372	-81.6856643	3-8
		5	12.5444987	-81.690854	15-30
		6	12.525167	-81.74137	8-15
		7	12.54449873	-81.690854	3-8
		8	12.528351	-81.738108	8-15
		9	12.502166	-81.737508	3-8
		10	12.561211	-81.743368	8-15
		11	12.532223	-81.694101	8-15
		12	12.585002	-81.727575	8-15
		13	12.596227	-81.712812	15-30
		14	12.501147	-81.73847	15-30
		15	12.49461	-81.74465	8-15
		16	12.52477	-81.734179	3-8
		1	12.1638861	-81.8586056	8-15
		2	12.1547194	-81.8483306	8-15
		3	12.1958278	-81.8613861	3-8
		4	12.1861111	-81.8605528	3-8
		5	12.1372167	-81.8338889	8-15
		6	12.17555	-81.8486111	15-30
		7	12.1397194	-81.8638889	15-30

	Albuquerque (ALB)	8	12.16788	-81.86505	3-8
		9	12.16297	-81.84648	3-8
		10	12.1377722	-81.8711083	8-15
		11	12.18838833	-81.8833306	15-30
		12	12.1649972	-81.8958306	15-30
		13	12.1436083	-81.8369417	8-15
		14	12.1647194	-81.8266667	15-30
		15	12.1525	-81.89223	3-8
		16	12.14083	-81.82389	15-30
	Providencia (PRO)	1	13.318977	-81.372128	8-15
		2	13.31263	-81.377107	8-15
		3	13.3143	-81.386891	3-8
		4	13.314634	-81.413499	3-8
		5	13.326494	-81.423455	8-15
		6	13.334178	-81.412812	15-30
		7	13.34921	-81.412984	15-30
		8	13.358731	-81.403542	8-15
		9	13.3662906	-81.413327	15-30
		10	13.374931	-81.412125	8-15
		11	13.379941	-81.410752	15-30
		12	13.387456	-81.400967	3-8
		13	13.393968	-81.393414	3-8
		14	13.393968	-81.351014	3-8
		15	13.403821	-81.378651	15-30
		16	13.397141	-81.381913	15-30
2019	San Andrés (SA)	1	12.502166	-81.737508	8-15
		2	12.501147	-81.73847	8-15
		3	12.561211	-81.743368	8-15
		4	12.49461	-81.74465	8-15
		5	12.562823	-81.700205	3-8*
		6	12.559254	-81.700737	3-8*
		7	12.1649972	-81.8958306	15-30
		8	12.1397194	-81.8638889	15-30
		9	12.56185	-81.696415	3-8*
		10	12.567172	-81.727137	3-8*
		11	12.595958	-81.718196	15-30
		12	12.16788	-81.86505	3-8
		13	12.532223	-81.694101	8-15
		14	13.3143	-81.386891	3-8
		15	13.314634	-81.413499	3-8
		16	12.5444987	-81.690854	15-30
		1	13.314634	-81.413499	3-8
		2	13.374931	-81.412125	8-15
		3	13.3143	-81.386891	3-8

	Providencia (PRO)	4	13.318977	-81.372128	8-15
		5	13.3662906	-81.413327	15-30
		6	13.38186	-81.378145	3-8*
		7	13.31263	-81.377107	8-15
		8	13.381526	-81.373853	3-8*
		9	13.334178	-81.412812	15-30
		10	13.375347	-81.353425	3-8*
		11	13.326494	-81.423455	8-15
		12	13.358731	-81.403542	8-15
		13	13.387456	-81.400967	3-8
		14	13.403821	-81.378651	15-30
		15	13.393968	-81.351014	3-8
		16	13.397141	-81.381913	15-30
