# Supplementary material for: Spatial variation of parrotfish assemblages at oceanic islands in the western Caribbean: evidence of indirect effects of fishing?
Source: PeerJ. 2022 Nov 28;10:e14178. doi: 10.7717/peerj.14178 (PMC9744149; doi:10.7717/peerj.14178)
Supplement: Supplemental Information 2 — (A) Data set with Sex, Species and Locality as fixed factors. (B) Data set with Sex and Locality as fixed factors. (C) Data set with Sex, Species, Locality and Year as fixed factors. (D) Data with Species, Locality and Year as fixed factors. [file peerj-10-14178-s002.pdf]

Table S2. Mixed effects models applied on body-size data. (A) Data set with Sex, Species and Locality as fixed factors. (B) Data set with Sex and Locality as fixed factors. (C) Data set with Sex, Species, Locality and Year as fixed factors. (D) Data with Species, Locality and Year as fixed factors.

(A)

M.a <- formula(Size ~ Sex * Species * Locality)
M1 <- gls(M.a , method = "REML", data = PARROT)

Linear mixed-effects model fit by REML
Data: PARROT

AIC	BIC	logLik
53942.23	54216.51	-26929.11

Random effects:
Formula: ~1 | Station

	(Intercept)	Residual
StdDev:	17.00036	47.63831

Number of Observations: 5108
Number of Groups: 64

M2 <- lme (M.a, random = ~1 | Station, weights = varIdent(form = ~ 1 | Species), data = PARROT)

Linear mixed-effects model fit by REML
Data: PARROT

AIC	BIC	logLik
53245.79	53546.2	-26576.89

Random effects:
Formula: ~1 | Stations

	(Intercept)	Residual
StdDev:	14.5811	42.24512

Variance function:
Structure: Different standard deviations per stratum
Formula: ~1 | Species
Parameter estimates:

S. taeniopterus	*S. iseri*	*S. viride*	*S. vetula*	*S.* aurofrenatum
1.00	0.80	1.70	1.50	1.04

Number of Observations: 5108
Number of Groups: 64

	Model	df	AIC	BIC	logLik	Test	L.Ratio	p-value
M1	1	44	53837.26	54124.61	-26874.63			
M2	2	46	53245.79	53546.20	-26576.89	1 vs 2	595.4719	<.0001

GAMMs

M3 <- gamm(Size ~ Sex * Species * Locality, random = list(Station =~ 1), data = PARROT)

	Length	Class	Mode
lme	18	Lme	List
gam	30	30	List

anova(M3$gam)

Family: gaussian
Link function: identity

Formula:
Size ~ Species * Locality * Sex

Parametric Terms:

	df	F	p-value
Species	4	79.418	< 2e-16
Locality	3	3.124	0.0248
Sex	1	49.549	2.19e-12
Species:Locality:Sex	12	5.343	4.60e-09

R-sq.(adj) = 0.571
Scale est. = 2253.4   n = 5108

(B)

M.b <- formula(Size ~ Species * Locality)
M4 <- gls(M.b , method = "REML", data = PARROT1)

Linear mixed-effects model fit by REML
Data: PARROT1

AIC	BIC	logLik
63974.98	64201.62	-31953.49

Random effects:
Formula: ~1 | Stations

	(Intercept)	Residual
StdDev:	19.50081	58.20187

Number of Observations: 5834
Number of Groups: 64

M5<- lme(M.b , random = ~ 1 | Station, weights = varIdent(form = ~ 1 | Species), data = PARROT1)

Linear mixed-effects model fit by REML
Data: PARROT1

AIC	BIC	logLik
62702.3	62975.61	-31310.15

Random effects:
 Formula: ~1 | Station

	(Intercept)	Residual
StdDev:	16.80161 5	0.75933

Variance function:
 Structure: Different standard deviations per stratum
 Formula: ~1 | Species
 Parameter estimates:

S. taeniopterus	*S. iseri*	*S. viride*	*S. vetula*	*S. aurofrenatum*	*S. rubripinne*	*S.* chrysopterum
1.00	0.78	1.89	1.56	1.08	1.52	1.44

Number of Observations: 5834
Number of Groups: 64

	Model	df	AIC	BIC	logLik	Test	L.Ratio	p-value
M1	1	33	64291.13	64511.10	-32112.56			
M2	2	41	62702.30	62975.61	-31310.15	1 vs 2	1604.821	<.0001

GAMMs

Family: gaussian
Link function: identity

Formula:
Size ~ Species * Locality

M6 <- gamm(Size ~ Species * Locality, random = list(Station =~ 1), data = PARROT1)

anova(M6$gam)
Parametric Terms:

	df	F	p-value
Species	7	108.053	< 2e-16
Locality	3	2.866	0.0352
Species:Locality	21	12.207	< 2e-16

R-sq.(adj) = 0.396
 Scale est. = 3371.4   n = 5834

(C)

M.f <- formula(Size ~ Species * Locality*Year*Sex)
M7 <- gls(M.e , method = "REML", data = PARROT3)

Linear mixed-effects model fit by REML
 Data: PARROT3

AIC	BIC	logLik
60855.33	61134.92	-30385.67

Random effects:
Formula: ~1 | Stations

	(Intercept)	Residual
StdDev:	13.65538	46.53488

Number of Observations: 5789
Number of Groups: 64

M8 <- lme(M.e , random = ~ 1 | Station, weights = varIdent(form = ~ 1 | Species), data = PARROT3)

Linear mixed-effects model fit by REML
Data: PARROT3

AIC	BIC	logLik
60228.06	60534.27	-30068.03

Random effects:
Formula: ~1 | Station

	(Intercept)	Residual
StdDev:	13.5467	39.13895

Variance function:
Structure: Different standard deviations per stratum
Formula: ~1 | Species
Parameter estimates:

S. taeniopterus	*S. iseri*	*S. viride*	*S. aurofrenatum*	*S. vetula*
1.00	0.90	1.67	1.14	1.66

Number of Observations: 5789
Number of Groups: 64

	Model	df	AIC	BIC	logLik	Test	L.Ratio	p-value
M7	1	41	61142.86	61415.79	--30530.43			
M8	2	46	60228.06	60534.27	-30068.03	1 vs 2	924.8064	<.0001

GAMMs

anova(M9$gam)

Parametric Terms:

	df	F	p-value
Species	4	5.458	0.000220
Locality	1	0.002	0.0248
Sex	1	5.601	2.19e-12
Year	1	1.165	0.280405
Species:Locality:Sex:Year	4	5.675	0.294356

R-sq.(adj) = 0.342
Scale est. = 3461.9 n = 6253

(D)

```
M.e <- formula(Size ~ Species * Locality*Year)
M10 <- gls(M.e , method = "REML", data = PARROT4)

Linear mixed-effects model fit by REML
 Data: PARROT4
      AIC         BIC          logLik
  68738.95    68941.04    -34339.48

Random effects:
 Formula: ~1 | Stations
              (Intercept)     Residual
StdDev:    16.48995     58.94914

Number of Observations: 6253
Number of Groups: 64

M11 <- lme(M.e , random = ~ 1 | Station, weights = varIdent(form = ~ 1 | Species), data =
PARROT4)

Linear mixed-effects model fit by REML
 Data: PARROT4
      AIC          BIC          logLik
  68032.83    68275.33    -33980.41

Random effects:
 Formula: ~1 | Stations
               (Intercept)     Residual
StdDev:    15.99506       49.60637

Variance function:
 Structure: Different standard deviations per stratum
 Formula: ~1 | Species
 Parameter estimates:
```

S. taeniopterus	*S. iseri*	*S. viride*	*S. aurofrenatum*	*S. vetula*	*S. chrysopterum*	*S. rubripinne*
1.00	0.88	1.66	1.07	1.56	1.44	1.62

```
Number of Observations: 6253
Number of Groups: 64
```

	Model	df	AIC	BIC	logLik	Test	L.Ratio	p-value
M10	1	29	69003.32	69198.67	-34472.66			
M11	2	36	68032.83	68275.33	-33980.41	1 vs 2	984.4941	<.0001

GAMMs

```
M12 <- gamm(Size ~  Species * Locality * Year, random = list(Station =~ 1), data = PARROT4)

anova(M3$gam)

Family: gaussian
Link function: identity

Formula:
Size ~ Species * Locality * Year

Parametric Terms:
                        df    F       p-value
  Species               6     7.447   5.79e-08
  Locality              1     1.451   0.988
  Year                  1     1.861   0.173
  Species:Locality:Year 6     1.449   0.192

R-sq.(adj) =  0.342
  Scale est. = 3461.9    n = 6253
```
