# Supplementary material for: Spatial variation of parrotfish assemblages at oceanic islands in the western Caribbean: evidence of indirect effects of fishing?
Source: PeerJ. 2022 Nov 28;10:e14178. doi: 10.7717/peerj.14178 (PMC9744149; doi:10.7717/peerj.14178)
Supplement: Supplemental Information 3 — Sample size (n) of populations whose TL was calculated; mean, minimun and maximun TL for each species and their males (M) and females (F) populations is presented for San Andrés (SA), Bolivar (BOL), Albuquerque (ALB), and Providencia and Santa catalina (PRO). [file peerj-10-14178-s003.pdf]

Table S3. Summary of total length (TL) results collected in 2018. Sample size (n) of populations whose TL was calculated; mean, minimun and maximun TL for each species and their males (M) and females (F) populations is presented for San Andrés (SA), Bolivar (BOL), Albuquerque (ALB), and Providencia and Santa catalina (PRO).

	**SA**				**BOL**				**ALB**				**PRO**			
**SPECIE**	**n**	**MEAN (mm)**	**Min. (mm)**	**Max. (mm)**	**n**	**MEAN (mm)**	**Min. (mm)**	**Max. (mm)**	**n**	**MEAN (mm)**	**Min. (mm)**	**Max. (mm)**	**n**	**MEAN (mm)**	**Min. (mm)**	**Max. (mm)**
Scarus coelestinus	2	574	487	660	3	510	273	697	4	333	268	420	10	479	387	597
Scarus coeruleus													9	548	452	600
(F)																
(M)													9	548	452	600
Scarus guacamaia													2	497	479	515
(F)													2	497	479	515
(M)																
Scaris iseri/taeniopterus	115	89	28	213	122	95	35	273	268	100	29	270	111	78	28	195
Scarus iseri	383	127	23	253	441	116	28	274	186	125	40	228	484	109	35	279
(F)	324	116	23	253	363	107	28	252	104	105	40	228	362	97	35	279
(M)	59	187	88	239	78	159	82	274	82	150	95	215	122	146	83	245
Scarus taeniopterus	543	148	36	369	269	151	26	280	165	158	47	289	311	139	34	352
(F)	450	137	36	369	228	140	26	249	135	144	47	243	258	122	34	352
(M)	93	202	112	317	41	216	87	280	30	222	130	289	53	222	84	326
Scarus vetula	59	212	75	526	77	228	71	394	66	219	91	370	55	260	81	462
(F)	52	200	75	526	57	189	71	350	54	202	91	314	40	230	81	367
(M)	7	301	238	417	20	340	270	394	12	300	224	370	15	340	292	462
Sparisoma atomarium	48	87	41	188	42	76	31	144	26	87	54	350	45	94	42	360
Sparisoma aurofrenatum	352	149	26	255	289	140	26	261	233	152	34	319	364	165	29	331
(F)	289	136	26	239	238	122	26	239	175	127	34	231	272	149	29	331
(M)	63	210	178	255	51	220	187	261	58	226	187	319	92	210	171	263
Sparisoma chrysopterum	9	301	198	375	5	307	118	414	6	339	315	388	29	293	59	365
Sparisoma rubripinne	7	278	174	337	19	294	131	385	27	239	85	365	8	308	263	342
Sparisoma viride	140	185	25	421	203	229	40	441	216	246	41	549	272	261	25	423
(F)	124	167	25	334	161	190	40	366	153	202	41	523	201	227	25	377
(M)	16	326	220	421	42	375	236	441	63	354	191	549	71	357	271	423
