# Supplementary material for: Spatial variation of parrotfish assemblages at oceanic islands in the western Caribbean: evidence of indirect effects of fishing?
Source: PeerJ. 2022 Nov 28;10:e14178. doi: 10.7717/peerj.14178 (PMC9744149; doi:10.7717/peerj.14178)
Supplement: Supplemental Information 4 — Sample size (n) of populations whose TL was calculated; mean, minimun and maximun TL for each species and their males (M) and females (F) populations is presented for San Andrés (SA), and Providencia and Santa catalina (PRO). [file peerj-10-14178-s004.pdf]

Table S4. Summary of total length (TL) results collected in 2019. Sample size (n) of populations whose TL was calculated; mean, minimun and maximun TL for each species and their males (M) and females (F) populations is presented for San Andrés (SA), and Providencia and Santa catalina (PRO).

	SA				PRO			
**SPECIES (SEX)**	**n**	**MEAN (mm)**	**Min. (mm)**	**Max. (mm)**	**n**	**MEAN (mm)**	**Min. (mm)**	**Max. (mm)**
Scarus coelestinus	22	533	234	903	31	393	214	640
Scarus coeruleus	1	348	348	348	5	189	86	280
Scarus guacamaia	23	351	207	1042	20	311	154	701
(F)	18	294	207	519	17	261	154	514
(M)	5	556	383	1042	3	595	531	701
Scaris iseri/taeniopterus	272	71	16	200	132	62	30	145
Scarus iseri	439	138	29	282	278	118	41	232
(F)	353	123	29	246	207	101	41	203
(M)	86	199	65	282	71	166	124	232
Scarus taeniopterus	630	159	49	345	43	159	78	294
(F)	501	142	49	296	33	130	78	238
(M)	129	224	140	345	10	252	233	294
Scarus vetula	153	182	42	418	153	220	40	352
(F)	141	169	42	403	121	199	40	306
(M)	12	326	240	418	32	298	221	352
Sparisoma atomarium	13	95	55	138	7	72	36	112
Sparisoma aurofrenatum	423	158	26	287	233	154	27	268
(F)	323	141	26	271	202	142	27	266
(M)	100	214	165	287	31	231	162	268
Sparisoma chrysopterum	85	223	85	356	118	250	91	431
(F)	51	190	85	343	77	236	91	429
(M)	34	272	187	356	41	276	179	431
Sparisoma rubripinne	55	209	73	359	153	253	68	463
Sparisoma viride	274	192	31	530	200	251	45	439
(F)	231	173	31	530	139	217	45	347
(M)	43	299	160	425	61	330	179	439
