# Supplementary material for: Spatial variation of parrotfish assemblages at oceanic islands in the western Caribbean: evidence of indirect effects of fishing?
Source: PeerJ. 2022 Nov 28;10:e14178. doi: 10.7717/peerj.14178 (PMC9744149; doi:10.7717/peerj.14178)
Supplement: Supplemental Information 5 — Average abundance (with square root tranformation) of species between location were significant differences were found, the average dissimilary (Av.Diss), their contribution (Contrib%) to the within-group disssimilarity, and the cumulative total (Cum.%) of contributions (90% cut-off). Sex is detailed in the corresponding SIMPER summary (M: Males; F: Females) [file peerj-10-14178-s005.pdf]

Table S5. Summary of SIMPER results for parrotfishes abundances data collected in 2018. Average abundance (with square root tranformation) of species between location were significant differences were found, the average dissimilary (Av.Diss), their contribution (Contrib%) to the within-group disssimilarity, and the cumulative total (Cum.%) of contributions (90% cut-off). Sex is detailed in the corresponding SIMPER summary (M: Males; F: Females)

	**SPECIES ABUNDANCE 2018**					
	Average abundance		Av. Diss	SD	Contrib%	Cum.%
**Locality**	**ALB**	**SA**				
Scarus iseri	4	6	6	1	21	21
Scarus taeniopterus	4	6	6	1	20	41
Sparisoma aurofrenatum	4	5	4	1	13	54
Sparisoma viride	4	3	4	1	12	66
Scarus vetula	2	2	3	1	11	77
Sparisoma atomarium	1	2	3	1	9	86
Sparisoma rubripinne	1	0	2	1	8	94
	**BOL**	**SA**				
Scarus taeniopterus	4	6	7	1	24	24
Scarus iseri	5	6	5	1	17	41
Scarus vetula	2	2	3	1	13	54
Sparisoma viride	4	3	3	1	12	66
Sparisoma aurofrenatum	5	5	3	1	11	77
Sparisoma atomarium	1	2	3	1	11	88
Sparisoma rubripinne	1	0	2	1	6	94
	**SA**	**PRO**				
Scarus taeniopterus	6	5	5	1	21	21
Scarus iseri	6	5	4	1	17	38
Sparisoma viride	3	4	4	1	14	53
Scarus vetula	2	2	3	1	12	65
Sparisoma aurofrenatum	5	5	3	1	10	75
Sparisoma atomarium	2	2	3	1	10	85
Sparisoma chrysopterum	0	1	2	1	6	92
	**SPECIES (SEX) ABUNDANCE 2018**					
	**ALB**	**SA**				
Scarus iseri (F)	3	5	5	1	15	15
Scarus taeniopterus (F)	4	5	5	1	14	29
Sparisoma aurofrenatum (F)	4	5	3	1	9	38
Sparisoma viride (F)	3	3	3	1	8	47
Scarus taeniopterus (M)	1	2	3	2	8	55
Scarus vetula (F)	2	2	3	1	7	62
Sparisoma atomarium	1	2	2	1	7	69
Sparisoma viride (M)	2	1	2	1	7	76
Scarus iseri (M)	2	2	2	1	6	82
Sparisoma rubripinne	1	0	2	1	6	88
Sparisoma aurofrenatum (M)	2	2	1	1	4	92
	**BOL**	**SA**				
Scarus taeniopterus (F)	4	5	5	1	17	17
Scarus iseri (F)	5	5	4	1	13	29
Scarus vetula (F)	2	2	3	1	9	38
Sparisoma viride (F)	3	3	3	1	9	47
Scarus taeniopterus (M)	1	2	2	2	8	55
Sparisoma atomarium	1	2	2	1	8	63
Sparisoma aurofrenatum (F)	4	5	2	1	8	71
Sparisoma viride (M)	1	1	2	1	6	76
Scarus vetula (M)	1	0	2	1	5	82

Scarus iseri (M)	2	2	1	1	5	86
Sparisoma aurofrenatum (M)	2	2	1	1	5	91
	**SA**	**PRO**				
Scarus taeniopterus (F)	5	4	4	1	14	14
Scarus iseri (F)	5	5	3	1	12	26
Sparisoma viride (F)	3	4	3	1	9	35
Sparisoma viride (M)	1	2	2	1	8	43
Scarus taeniopterus (M)	2	1	2	1	8	51
Scarus vetula (F)	2	1	2	1	8	59
Sparisoma atomarium	2	2	2	1	7	66
Sparisoma aurofrenatum (F)	5	4	2	1	7	73
Scarus iseri (M)	2	3	2	1	6	80
Sparisoma aurofrenatum (M)	2	3	1	1	5	85
Scarus vetula (M)	0	1	1	1	5	90
Sparisoma chrysopterum	0	1	1	1	5	94
