# Supplementary material for: Spatial variation of parrotfish assemblages at oceanic islands in the western Caribbean: evidence of indirect effects of fishing?
Source: PeerJ. 2022 Nov 28;10:e14178. doi: 10.7717/peerj.14178 (PMC9744149; doi:10.7717/peerj.14178)
Supplement: Supplemental Information 6 — Average abundance (with square root tranformation) of species between location were significant differences were found, the average dissimilary (Av.Diss), their contribution (Contrib%) to the within-group disssimilarity, and the cumulative total (Cum.%) of contributions (90% cut-off). Sex is detailed in the corresponding SIMPER summary (M: Males; F: Females) [file peerj-10-14178-s006.pdf]

Table S6. Summary of SIMPER results for parrotfishes abundances data collected in 2019. Average abundance (with square root tranformation) of species between location were significant differences were found, the average dissimilary (Av.Diss), their contribution (Contrib%) to the within-group disssimilarity, and the cumulative total (Cum.%) of contributions (90% cut-off). Sex is detailed in the corresponding SIMPER summary (M: Males; F: Females)

	Average abundance		Av. Diss	SD	Contrib%	Cum.%
	**SPECIES ABUNDANCE 2019**					
**Locality**	**PRO**	**SA**				
Scarus taeniopterus	1	6	11	2	25	25
Scarus iseri	5	6	5	1	13	38
Scarus vetula	2	3	5	1	11	49
Sparisoma rubripinne	2	1	4	1	11	59
Sparisoma chrysopterum	2	2	4	1	10	70
Sparisoma aurofrenatum	4	5	4	1	10	80
Sparisoma viride	3	4	3	1	7	87
Scarus coelestinus	1	0	2	1	5	92
	**SPECIES ABUNDANCE (SEX) 2019**					
Scarus taeniopterus (F)	1	6	8	2	17	17
Scarus iseri (F)	4	5	4	1	9	27
Scarus taeniopterus (M)	0	2	4	1	8	35
Sparisoma rubripinne	2	1	4	1	8	43
Sparisoma chrysopterum	2	2	4	1	8	51
Scarus vetula (F)	2	2	4	1	8	59
Sparisoma aurofrenatum (F)	3	4	3	1	6	66
Sparisoma viride (F)	3	4	3	1	6	72
Sparisoma aurofrenatum (M)	1	2	2	2	6	77
Scarus iseri (M)	2	2	2	1	5	82
Sparisoma viride (M)	2	1	2	1	4	86
Scarus coelestinus	1	0	2	1	4	90
Scarus vetula (M)	1	1	2	1	4	93
