# Supplementary material for: Spatial variation of parrotfish assemblages at oceanic islands in the western Caribbean: evidence of indirect effects of fishing?
Source: PeerJ. 2022 Nov 28;10:e14178. doi: 10.7717/peerj.14178 (PMC9744149; doi:10.7717/peerj.14178)
Supplement: Supplemental Information 7 — Average abundance (estandarized biomass) of species between location were significant differences were found, the average dissimilary (Av.Diss), their contribution (Contrib%) to the within-group disssimilarity, and the cumulative total (Cum.%) of contributions (90% cut-off). Sex is detailed in the corresponding SIMPER summary (M: Males; F: Females) [file peerj-10-14178-s007.pdf]

Table S7. Summary of SIMPER results for parrotfishes biomass data collected in 2018. Average abundance (estandarized biomass) of species between location were significant differences were found, the average dissimilary (Av.Diss), their contribution (Contrib%) to the within-group disssimilarity, and the cumulative total (Cum.%) of contributions (90% cut-off). Sex is detailed in the corresponding SIMPER summary (M: Males; F: Females)

	**SPECIES BIOMASS 2018**					
	Average abundance		Av. Diss	SD	Contrib%	Cum.%
**Locality**	**ALB**	**SA**				
Scarus taeniopterus	0.8	0.48	6.65	1.59	17.98	17.98
Sparisoma viride	0.65	0.45	5.92	1.51	16	33.98
Scarus vetula	0.23	0.2	4.96	1.35	13.41	47.39
Scarus iseri	0.21	0.33	3.47	1.32	9.39	56.78
Sparisoma rubripinne	0.13	0.06	3.34	0.87	9.03	65.82
Sparisoma aurofrenatum	0.36	0.43	3.27	1.17	8.85	74.67
Sparisoma chrysopterum	0.09	0.12	3.21	1.05	8.67	83.34
Scarus iseri/taeniopterus	0.17	0.08	3.11	1.34	8.4	91.74
	**BOL**	**SA**				
Scarus vetula		0.2	6	1.36	17.62	17.62
Scarus taeniopterus	0.32	0.48	5.9	1.47	17.32	34.94
Sparisoma viride	0.61	0.45	5.19	1.47	15.24	50.18
Sparisoma aurofrenatum	0.33	0.43	3.28	1.28	9.63	59.82
Scarus coelestinus	0.07	0.06	2.8	0.43	8.23	68.05
Sparisoma chrysopterum	0.04	0.12	2.74	0.99	8.05	76.1
Scarus iseri	0.29	0.33	2.65	1.34	7.77	83.87
Sparisoma rubripinne	0.09	0.06	2.57	0.76	7.55	91.42
	**SA**	**PRO**				
Scarus taeniopterus	0.48	0.26	5.92	1.75	15.82	15.82
Scarus vetula	0.2	0.22	4.84	1.37	12.93	28.75
Sparisoma viride	0.45	0.64	4.81	1.31	12.86	41.6
Scarus coelestinus	0.06	0.13	3.81	0.61	10.2	51.8
Sparisoma aurofrenatum	0.43	0.35	3.54	1.32	9.46	61.26
Sparisoma chrysopterum	0.12	0.1	3.48	0.85	9.3	70.55
Scarus iseri	0.33	0.22	3.08	1.46	8.23	78.78
Scarus coeruleus	0	0.14	3.04	0.54	8.12	86.9
Sparisoma rubripinne	0.06	0.04	1.81	0.68	4.85	91.75
	**SPECIES (SEX) BIOMASS 2018**					
	**ALB**	**SA**				
Sparisoma viride (M)	0.43	0.23	5.9	1.49	14.5	14.5
Scarus taeniopterus (F)	0.21	0.39	4.57	1.7	11.23	25.74
Scarus taeniopterus (M)	0.16	0.29	3.7	1.37	9.09	34.83
Sparisoma viride (F)	0.43	0.37	3.4	1.3	8.38	43.21
Scarus vetula (F)	0.2	0.16	3.39	1.2	8.35	51.56
Scarus iseri (F)	0.11	0.27	3.16	1.64	7.77	59.33
Sparisoma aurofrenatum (F)	0.24	0.36	2.84	1.24	6.99	66.32
Sparisoma rubripinne	0.13	0.06	2.7	0.87	6.64	72.96
Sparisoma chrysopterum	0.09	0.12	2.58	1.05	6.35	79.31
Scarus iseri/taeniopterus	0.17	0.08	2.53	1.35	6.22	85.54
Sparisoma aurofrenatum (M)	0.26	0.27	2.04	1.28	5.02	90.56
	**BOL**	**SA**				
Sparisoma viride (M)	0.34	0.23	4.92	1.4	13.63	13.63
Scarus taeniopterus (F)	0.24	0.39	4.2	1.4	11.63	25.26
Scarus vetula (M)	0.21	0.07	3.77	1.09	10.44	35.7
Scarus vetula (F)	0.17	0.16	3.34	1.2	9.26	44.96
Scarus taeniopterus (M)	0.2	0.29	3.19	1.5	8.84	53.8

Sparisoma viride (F)	0.46	0.37	3.15	1.21	8.73	62.53
Sparisoma aurofrenatum (F)	0.25	0.36	2.37	1.26	6.57	69.1
Sparisoma chrysopterum	0.04	0.12	2.17	0.99	6.02	75.12
Sparisoma rubripinne	0.09	0.06	2.14	0.72	5.94	81.06
Scarus iseri (F)	0.22	0.27	1.95	1.23	5.41	86.46
Sparisoma aurofrenatum (M)	0.23	0.27	1.69	1.34	4.69	91.15
	**SA**	**PRO**				
Sparisoma viride (M)	0.23	0.47	5.01	1.42	14.43	14.43
Scarus taeniopterus (F)	0.39	0.21	3.99	1.76	11.49	25.92
Sparisoma viride (F)	0.37	0.49	3.71	1.35	10.66	36.58
Scarus taeniopterus (M)	0.29	0.18	3.08	1.4	8.87	45.46
Scarus vetula (F)	0.16	0.16	3.08	1.16	8.86	54.31
Sparisoma chrysopterum	0.12	0.11	2.96	0.87	8.53	62.84
Scarus vetula (M)	0.07	0.15	2.81	1.1	8.08	70.93
Sparisoma aurofrenatum (F)	0.36	0.28	2.35	1.22	6.78	77.7
Scarus iseri (F)	0.27	0.18	1.88	1.28	5.42	83.12
Sparisoma aurofrenatum (M)	0.27	0.25	1.66	1.33	4.77	87.88
Sparisoma rubripinne	0.06	0.05	1.55	0.68	4.45	92.33
