# Supplementary material for: Spatial variation of parrotfish assemblages at oceanic islands in the western Caribbean: evidence of indirect effects of fishing?
Source: PeerJ. 2022 Nov 28;10:e14178. doi: 10.7717/peerj.14178 (PMC9744149; doi:10.7717/peerj.14178)
Supplement: Supplemental Information 8 — Average abundance (estandarized biomass) of species between location were significant differences were found, the average dissimilary (Av.Diss), their contribution (Contrib%) to the within-group disssimilarity, and the cumulative total (Cum.%) of contributions (90% cut-off). Sex is detailed in the corresponding SIMPER summary (M: Males; F: Females) [file peerj-10-14178-s008.pdf]

Table S8. Summary of SIMPER results for parrotfishes biomass data collected in 2019. Average abundance (estandarized biomass) of species between location were significant differences were found, the average dissimilary (Av.Diss), their contribution (Contrib%) to the within-group disssimilarity, and the cumulative total (Cum.%) of contributions (90% cut-off). Sex is detailed in the corresponding SIMPER summary (M: Males; F: Females)

	**SPECIES BIOMASS 2019**					
	Average abundance		Av. Diss	SD	Contrib%	Cum.%
**Locality**	**PRO**	**SA**				
Scarus taeniopterus	0.08	0.39	7.52	1.56	15.95	15.95
Sparisoma chrysopterum	0.25	0.2	6.27	1.06	13.29	29.24
Sparisoma rubripinne	0.27	0.09	5.66	1.26	11.99	41.24
Scarus coelestinus	0.22	0.05	5.19	0.79	11.01	52.24
Scarus vetula	0.21	0.26	5.13	1.35	10.87	63.11
Sparisoma viride	0.56	0.45	4.34	1.29	9.21	72.32
Sparisoma aurofrenatum	0.28	0.36	3.83	1.25	8.13	80.45
Scarus iseri	0.18	0.29	3.71	1.37	7.87	88.32
Scarus guacamaia	0.1	0.09	3.18	0.74	6.74	95.06
	**SPECIES (SEX) BIOMASS 2019**					
Sparisoma chrysopterum	0.26	0.22	5.4	1.1	12.22	12.22
Sparisoma rubripinne	0.29	0.11	5.19	1.27	11.75	23.97
Scarus taeniopterus (F)	0.07	0.29	4.47	1.59	10.1	34.08
Scarus taeniopterus (M)	0.07	0.27	4.46	1.39	10.09	44.16
Sparisoma viride (M)	0.41	0.27	4.08	1.23	9.22	53.38
Scarus vetula (F)	0.17	0.23	3.71	1.35	8.39	61.77
Sparisoma viride (F)	0.41	0.38	3.18	1.23	7.2	68.97
Scarus vetula (M)	0.13	0.11	2.83	1.08	6.41	75.38
Sparisoma aurofrenatum (M)	0.17	0.25	2.79	1.37	6.3	81.68
Scarus iseri (F)	0.13	0.22	2.42	1.3	5.47	87.15
Sparisoma aurofrenatum (F)	0.25	0.26	2.39	1.34	5.41	92.56
