# Supplementary figures and images for: Spatial variation of parrotfish assemblages at oceanic islands in the western Caribbean: evidence of indirect effects of fishing?
Source: PeerJ. 2022 Nov 28;10:e14178. doi: 10.7717/peerj.14178 (PMC9744149; doi:10.7717/peerj.14178)

A)

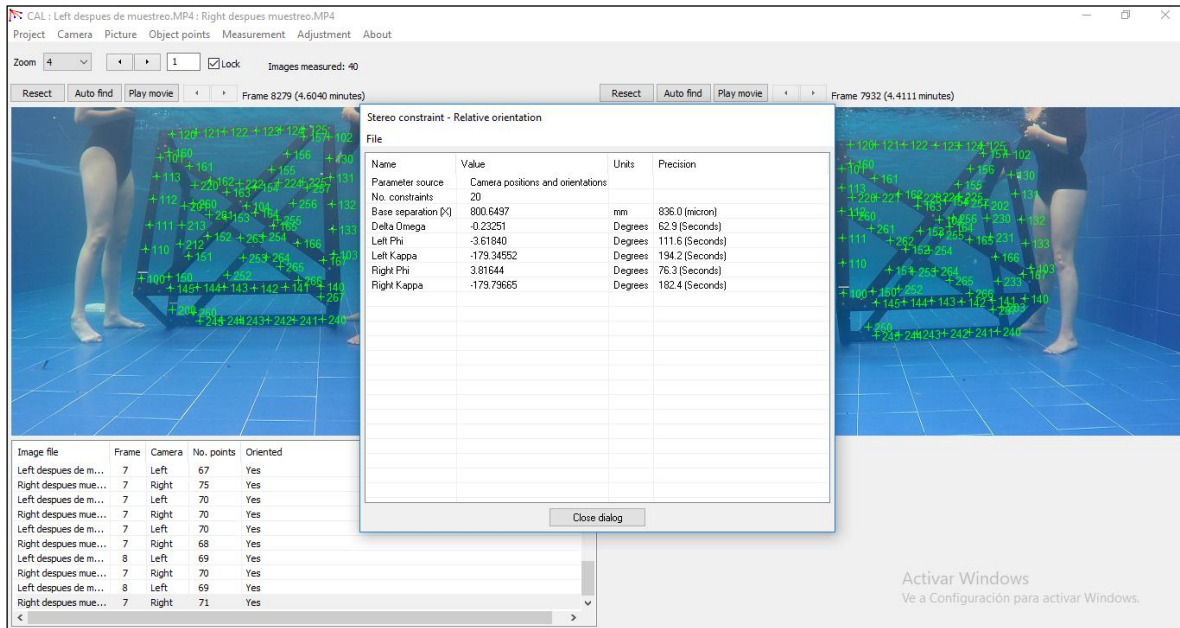

B)

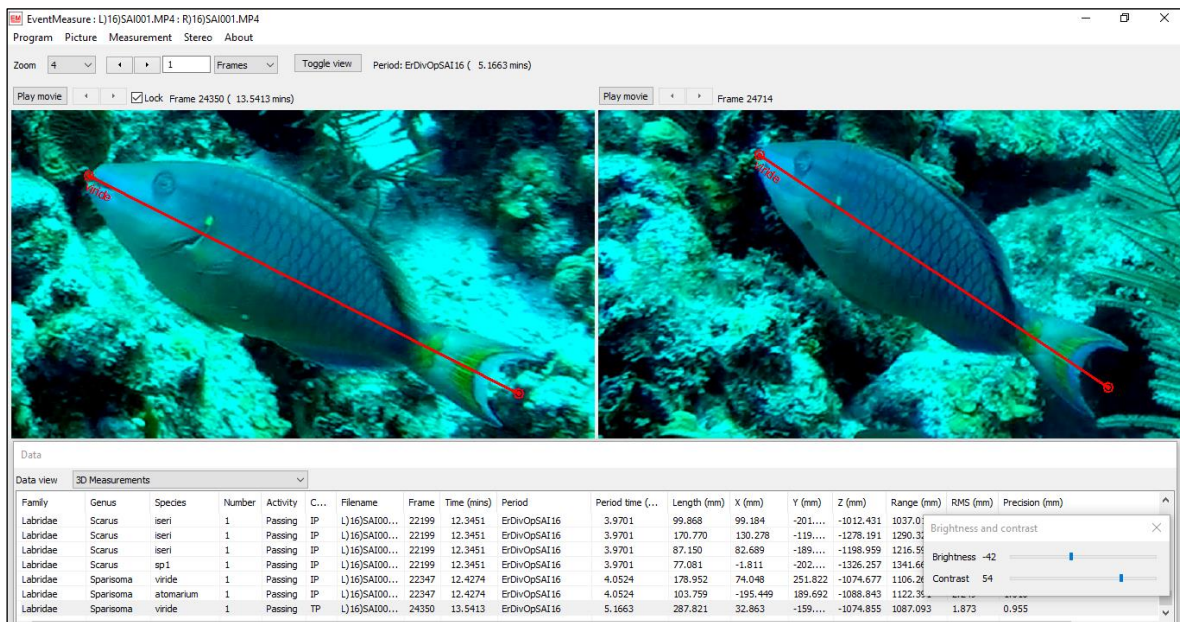

Supplement: Supplemental Information 9 — A. Calibration using the CAL software and a cube with approximately 80 targets. B. Total length calculation using the Event Measure software. SeaGIS Pty Ltd. Bacchus Marsh, Victoria, Australia [file peerj-10-14178-s009.pdf]
